# New deletion alleles for *Caenorhabditis elegans* Hedgehog pathway-related genes *wrt-6* and *wrt-10*

**DOI:** 10.17912/micropub.biology.000169

**Published:** 2019-10-15

**Authors:** Tessa Sherry, Hannah R Nicholas, Roger Pocock

**Affiliations:** 1 Development and Stem Cells Program, Monash Biomedicine Discovery Institute and Department of Anatomy and Developmental Biology, Monash University, Melbourne, Victoria 3800, Australia; 2 School of Life and Environmental Sciences, The University of Sydney, Sydney, NSW 2006, Australia

**Figure 1:**
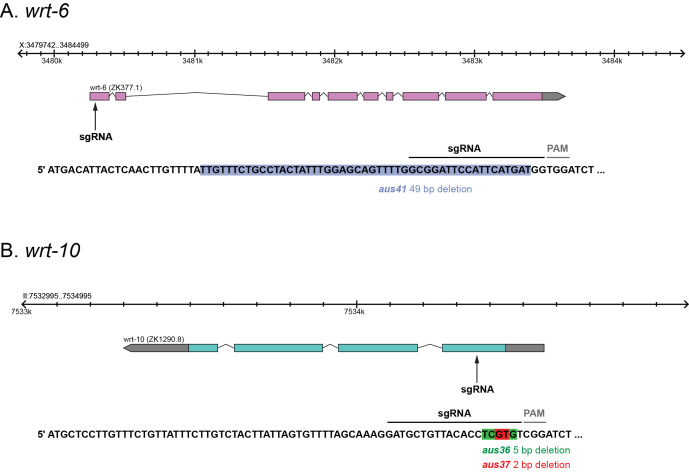
**Deletion alleles for *wrt-6* and *wrt-10*. A.** *wrt-6(aus41)*. **B.** *wrt-10(aus36)* and *wrt-10(aus37)*.

## Description

We have generated new putative null alleles of two members of the *C. elegans* Hedgehog-related pathway: *wrt-6* and *wrt-10*. The *warthog* (*wrt*) family member *wrt-6* encodes a predicted secreted signalling molecule that contains an N-terminal Wart domain and a C-terminal autoprocessing domain (Hint or Hog domain) (Bürglin 1996; Aspöck et al. 1999; Bürglin, 2008). *wrt-10* encodes another member of this Wrt family that contains the Wart domain but lacks the Hog domain (Bürglin 1996; Aspöck et al. 1999).

To generate deletion alleles, we used CRISPR-Cas9 to target the first exon of *wrt-6* or *wrt-10* (*wrt-6* sgRNA: 5′-GATGCTGTTACACCTCGTGT-3′, *wrt-10* sgRNA: 5′-GCGGATTCCATTCATGATGG-3′). We isolated the *wrt-6 (aus41)* allele that contains a 49 bp out-of-frame deletion, predicted to cause a premature stop codon after 7 amino acids (Figure 1A). We also generated two *wrt-10* deletion alleles: *aus36* and *aus37*. The *aus36* allele contains a 5 bp deletion, predicted to cause a premature stop codon after 33 amino acids (Figure 1B). The *aus37* allele contains a 2 bp deletion, predicted to cause a premature stop codon after 34 amino acids (Figure 1B). As all these alleles are predicted to cause premature stop codons in the first exon of each gene, they are likely to represent molecular nulls. We found that these alleles are viable and have no obvious gross morphological phenotype. Therefore, in-depth phenotypic examination is required to dissect their functional role. To conclude, we have isolated specific deletion alleles for *wrt-6* and *wrt-10* and will therefore help reveal new insights into the functions of Hedgehog-related signaling in *C. elegans*.

## Methods

sgRNA target sequences were incorporated into a *pU6::klp-12* sgRNA expression vector by PCR (Norris et al. 2015). Wild-type (N2) animals were injected with a mix consisting of the sgRNA expression vector (250 ng/µL), Cas9 expression vector (*peft-3::cas9::tbb-2*) (50 ng/µL) (gift from the de Bono Lab), pCFJ90 (*pmyo-2::mCherry::unc-54*) (2.5 ng/µL) and pCFJ104 (*pmyo-3::mCherry::unc-54*) (5 ng/µL). We used a 3-primer method for screening for deletions in mCherry-positive animals (external forward, internal forward directly upstream of PAM site, and external reverse; see Reagents for sequences). The external forward and reverse primers were used for Sanger sequencing.

## Reagents

**Oligonucleotides used to detect and genotype *wrt-6(aus41)***

Expected PCR amplicon: Wild-type: 578 and 327 bp. Mutant: 529 bp

External forward: GTGTCGTCGGATTTCTTCATTC

Internal forward: GCGGATTCCATTCATGATGG

External reverse: GAGCCACTTACAGTCCAAGG

**Oligonucleotides used to detect and genotype *wrt-10(aus36)* and *(aus37)***

Expected PCR amplicon: Wild-type: 539 and 241 bp. Mutant: 534 bp (*aus36)*, 537 bp (*aus37)*

External forward: GCAACAATCGGTCCGTCCAC

Internal forward: GATGCTGTTACACCTCGTGT

External reverse: GGTTTCAGCACGGCAAGAAG

**Strains**

HRN666 *wrt-10(aus36) II*

HRN667 *wrt-10(aus37) II*

HRN680 *wrt-6(aus41) X*

Strains have not been outcrossed. Will be available at the CGC.
